# Anti-cancer properties of cannflavin A and potential synergistic effects with gemcitabine, cisplatin, and cannabinoids in bladder cancer

**DOI:** 10.1186/s42238-022-00151-y

**Published:** 2022-07-22

**Authors:** Andrea M. Tomko, Erin G. Whynot, Denis J. Dupré

**Affiliations:** Faculty of Medicine, Department of Pharmacology, Dalhousie University, PO BOX 15 000, Sir Charles Tupper Medical Building, 5850 College St., Halifax, NS B3H 4R2 Canada

**Keywords:** Bladder cancer, Gemcitabine, Cisplatin, Cannabidiol, Δ^9^-Tetrahydrocannabinol, Cannabichromene, Cannabivarin, Cannflavin A, Apoptosis, Invasion

## Abstract

**Introduction:**

Several studies have shown anti-tumor effects of components present in cannabis in different models. Unfortunately, little is known about the potential anti-tumoral effects of most compounds present in cannabis in bladder cancer and how these compounds could potentially positively or negatively impact the actions of chemotherapeutic agents. Our study aims to evaluate the effects of a compound found in *Cannabis sativa* that has not been extensively studied to date, cannflavin A, in bladder cancer cell lines. We aimed to identify whether cannflavin A co-treatment with agents commonly used to treat bladder cancer, such as gemcitabine and cisplatin, is able to produce synergistic effects. We also evaluated whether co-treatment of cannflavin A with various cannabinoids could produce synergistic effects.

**Methods:**

Two transitional cell carcinoma cell lines were used to assess the cytotoxic effects of the flavonoid cannflavin A up to 100 μM. We tested the potential synergistic cytotoxic effects of cannflavin A with gemcitabine (up to 100 nM), cisplatin (up to 100 μM), and cannabinoids (up to 10 μM). We also evaluated the activation of the apoptotic cascade using annexin V and whether cannflavin A has the ability to reduce invasion using a Matrigel assay.

**Results:**

Cell viability of bladder cancer cell lines was affected in a concentration-dependent fashion in response to cannflavin A, and its combination with gemcitabine or cisplatin induced differential responses—from antagonistic to additive—and synergism was also observed in some instances, depending on the concentrations and drugs used. Cannflavin A also activated apoptosis via caspase 3 cleavage and was able to reduce invasion by 50%. Interestingly, cannflavin A displayed synergistic properties with other cannabinoids like Δ^9^-tetrahydrocannabinol, cannabidiol, cannabichromene, and cannabivarin in the bladder cancer cell lines.

**Discussion:**

Our results indicate that compounds from *Cannabis sativa* other than cannabinoids, like the flavonoid cannflavin A, can be cytotoxic to human bladder transitional carcinoma cells and that this compound can exert synergistic effects when combined with other agents. In vivo studies will be needed to confirm the activity of cannflavin A as a potential agent for bladder cancer treatment.

**Supplementary Information:**

The online version contains supplementary material available at 10.1186/s42238-022-00151-y.

## Introduction

Transitional cell carcinoma (TCC) accounts for more than 90% of all bladder cancers (Pons et al. 2011). The majority of newly diagnosed TCC are lower-grade, superficial non-muscle invasive tumors; however, tumors may recur in a number of patients, with worsening grade and stage (Bellmunt et al. 2020). Before the development of effective chemotherapy, the median survival range rarely exceeded 6 months, but advances in combination chemotherapy improved median survival times to 14 months. Systemic combination chemotherapy, such as the methotrexate, vinblastine, doxorubicin, and cisplatin (MVAC) regimen, has proven efficacy in bladder cancer, but toxicity is often observed (Chester et al. 2004; Li et al. 2005). Alternative strategies that improve survival outcomes or lead to similar survival benefits with reduced toxicity compared to the MVAC regimen are still needed. One example of this is gemcitabine-based therapy, which can be used as intravesical instillations with minimal bladder irritation, or as a systemic administration (Moore et al. 1997; Laufer et al. 2003). Additionally, gemcitabine-cisplatin combination therapy is effective and safe and is frequently used as first-line therapy against metastatic bladder cancer (Moore et al. 1999; von der Maase et al. 2000; Bellmunt et al. 2012) since its toxicity profile is low and the efficacy of the treatment remains similar to the MVAC regimen. Although these regimens are effective, co-medication with other drugs may further improve the outcomes of such therapy.

Smoking is a risk factor for the development of bladder cancer. A study on the effects of cannabis and/or tobacco use was performed where men were followed over an 11-year period. While consumption of tobacco was associated with an increased risk of bladder cancer, cannabis use alone was associated with a 45% reduction in bladder cancer incidence (Thomas et al. 2015). Chronic cannabis use leads to accumulation of several components of cannabis in the urine, which may reduce the potential for tumor development in the bladder and subsequently reduce bladder cancer incidence. Due to their cytotoxic activity, these same cannabis compounds could also potentially be used to eliminate bladder tumors therapeutically. Over 100 phytocannabinoids have been identified (Mehmedic et al. 2010), but Δ^9^-tetrahydrocannabinol (THC) and cannabidiol (CBD) are the most common cannabinoids produced in the *Cannabis* plant (de Meijer et al. 2003; Mechoulam 2005). Interestingly, studies have found that multiple compounds from cannabis inhibit tumor cell growth and induce apoptosis in various cancer cells (Blázquez et al. 2006, 2008; Guzmán et al. 2006; Carracedo et al. 2006; Javid et al. 2016; Blasco-Benito et al. 2018; Tomko et al. 2019), but little is known about their effects in bladder cancer. Recently, a study suggested that cannabis-derived cannabichromene (CBC) and ∆^9^-tetrahydrocannabinol displayed some synergy when used together in a model of urothelial cell carcinoma (Anis et al. 2021). Another study showed that cannabidiol (CBD) effectively inhibited growth and migration and induced apoptosis by inactivating the PI3K/AKT pathway in bladder cancer cell lines. The authors also showed that various CBD-loaded nanoparticles had the potential to significantly enhance the adhesion of CBD in the bladder wall and reduce potential damage caused by repeated perfusions and therefore improve long-term treatment (Chen et al. 2021). Our group also observed that cannabinoids can produce synergistic cytotoxic effects with gemcitabine and/or cisplatin in bladder cancer cell lines. Along with cannabinoids, cannabis also produces other compounds, including terpenes and flavonoids. Flavonoids belong to a class of phenolic compounds and are reported to be associated with numerous health benefits. Over 20 flavonoids have been identified in cannabis, including cannflavins which are uniquely found in cannabis (Erridge et al. 2020). Generally, flavonoids have shown potential as cytotoxic anti-cancer agents promoting apoptosis in cancer cells, but their oral bioavailability has limited their development into therapies. Due to the potential to medicate via bladder instillation, this may not necessarily be an issue for bladder cancer. Recently, a study examined the potential of a cannflavin B derivative (FBL-03G) for the treatment of pancreatic cancer. In vitro results showed an increase in apoptosis in pancreatic cancer cell lines treated with FBL-03G. In vivo, local and metastatic pancreatic tumor progression was delayed, leading to increased survival levels compared to control cohorts (Moreau et al. 2019). Little is known about the potential actions of other cannflavins in cancer. Further research is required to understand the effect of the numerous compounds present in cannabis to understand which ones exert superior cytotoxic effects and how they may affect current chemotherapeutic agents. Our study presents the results of the effects of cannflavin A alone or in presence of other cannabinoids, as well as gemcitabine, cisplatin, or the combination of cisplatin and gemcitabine together in bladder cancer cell lines.

## Materials and methods

### Drugs

Gemcitabine, cisplatin, Δ^9^-tetrahydrocannabinol, cannabidiol, orientin, quercetin, silymarin, vitexin, isovitexin, and luteolin were obtained from Millipore-Sigma (Oakville ON, CA). Cannflavin A, kaempferol, apigenin, cannabichromene, and cannabivarin were obtained from Cayman Chemical (Ann Arbour MI, USA).

### Cell culture

Human bladder transitional cell carcinoma T24 (ATCC® HTB4™) (ATCC, Manassas VA, USA) and TCCSUP (ATCC® HTB5™) (ATCC, Manassas VA, USA) and non-tumorigenic human bladder epithelial cells HBlEpC (938-05a) (Cell Applications Inc., San Diego CA, USA) were cultured in McCoy’s 5A, Eagle’s Minimum Essential Medium (Millipore-Sigma, Oakville ON, CA), and EpiVita basal medium in conjunction with human bladder epithelial growth supplement from (Cell Applications Inc., San Diego CA, USA) respectively, with 1% penicillin-streptomycin containing 10% fetal bovine serum (Gibco, Life Technologies, Walton MA, USA) at 37 °C, in a 5% CO_2_ atmosphere. It was demonstrated that in vitro models can adequately reproduce clinically relevant results and may be suitable to identify novel substances for the treatment of bladder cancer (Vallo et al. 2015).

### Cytotoxicity assays

Cells were seeded at 3000 cells/well in 96-well plates and grown for 24 h before adding drugs. Cells were treated with increasing concentrations of flavonoids, cannabinoids, gemcitabine, and/or cisplatin for 48 h. To assess viability, AlamarBlue® (Bio-Rad Laboratories, Hercules CA, USA) was added to each well and incubated for 4 h at 37 °C as per the manufacturer’s instructions. Fluorescence was measured following excitation at 540 nm, and emission was read at 590 nm with a Biotek Cytation 3. Data are expressed as the percentage of viable cells vs. vehicle treated cells, normalized as 100%, and represented as mean ± SEM. The *p* values were obtained from the data of at least three independent experiments.

### Cell lysis and western blotting

Cells were lysed with RIPA buffer (150 mM NaCl, 50 mM Tris–HCl pH 7.5, 1% NP40, 0.5% sodium deoxycholate, 0.1% sodium dodecyl sulfate, and 1 complete EDTA-free protease inhibitor cocktail tablet) (Roche, Laval QC, USA). BSA-coated beads (Protein A-Sepharose, Sigma-Aldrich Oakville ON, CA) and 10% DNase I (Sigma-Aldrich, Oakville ON, CA) were added to remove nucleic acid and organellar material from the sample. Lysates were mixed 50:50 with 2X Laemmli Buffer and 2-mercaptoethanol (Bio-Rad Laboratories, Hercules CA, USA). Samples were run on SDS–PAGE gels and transferred to nitrocellulose membranes before being blocked in a 10% skim milk powder/PBS solution for 60 min and incubated overnight at 4 °C with their respective primary antibodies (cleaved Caspase 3 conjugated to HRP (p11): sc-271759 from Santa Cruz Biotechnologies, Dallas TX, USA) (alpha tubulin (H-300): sc-5546 from Santa Cruz Biotechnologies, Dallas TX, USA). Membranes incubated with the alpha tubulin primary were incubated with secondary antibody for 1h (anti-rabbit IgG, HRP linked antibody (7074s) from Cell Signaling Technology, Whitby ON, CA). Chemiluminescence was performed on nitrocellulose membranes using Western Lightning® Plus-ECL Enhanced Chemiluminescence Substrate (PerkinElmer, Woodbridge ON, CA) before exposing them to X-ray film and development.

### Apoptosis assay

Cells were grown on glass coverslips in 6-well plates and then treated with methanol or 2.5 μM cannflavin A for 24 h. The Annexin V apoptosis detection kit (Santa Cruz Biotechnologies) was used to determine the rate of apoptosis. Cells were harvested and washed with PBS, then resuspended in Annexin V Assay Buffer following the manufacturer’s instructions. Cells were gently shaken in the dark with propidium iodide (PI) and Annexin V-FITC-conjugated stain for 20 min. Cells were then examined by fluorescence microscopy and at least 5 fields of view were recorded using an Olympus IX81 microscope equipped with a Photometrics coolSNAP HQ2 camera and an Excite series 120Q light source. Annexin V stain was excited at 488 nm and images were captured at 525 nm. PI was excited at 535 nm and images captured at 617 nm. Rates of early apoptosis were determined by dividing the number of cells that stained positive for Annexin-V divided by the total number of cells (Martin et al. 2019; Young et al. 2015).

### Autophagy assay

Cells were seeded at 3000 cells/well in 96-well plates and grown for 24 h before adding drugs. Cells were treated with 2.5 μM cannflavin A with or without inhibitors of autophagy (100 nM bafilomycin A or the combination of 10 μg/ml E-64d (aloxistatin) and 10 μg/ml (pepstatin A) for 24 h. To assess viability, AlamarBlue® (Bio-Rad Laboratories) was added to each well and incubated for 24 h at 37 °C as per the manufacturer’s instructions. Fluorescence was measured following excitation at 540 nm, and emission was read at 590 nm with a Biotek Cytation 3. Data are expressed as the percentage of viable cells vs. vehicle treated cells, normalized as 100%, and represented as mean ± SEM. The *p* values were obtained from the data of at least three independent experiments.

### Transwell migration

T24 cells were suspended in McCoy’s 5A medium with no FBS at a concentration of 1.5 × 10^5^ cells/mL. Two hundred and fifty microliters of 0.2% FBS medium containing vehicle control was added into the top portion of a transwell migration well that contains a polycarbonate membrane (Costar, Tewksbury MA, USA). In the bottom portion of the well, 700 μL of McCoy’s 5A medium containing 10% FBS was added to direct the migration. Cells were incubated at 37 °C under these conditions for 24 h. Following incubation, media and cells that did not migrate were removed with a dampened cotton swab. Cells were then fixed in methanol for 10 min and stained with 3.5 g/L crystal violet in 2% ethanol for 10 min. Wells were rinsed thoroughly with dH_2_O and left to dry overnight. Cells that migrated were counted with an Olympus CKX41 light microscope. The total number of cells that migrated under vehicle conditions served as 100% for invasion assay calculations.

### Matrigel invasion

Growth factor-reduced 8.0 micron Matrigel Invasion Chambers (Corning, Tewksbury MA, USA) were added to a 24-well plate. Matrigel invasion chambers were hydrated for 1 h at 37°C with 250 μL of McCoy’s 5A medium containing 0.2% FBS and penicillin-streptomycin. T24 cells were then seeded in McCoy’s 5A medium without FBS at a concentration of 150,000 cells/mL. Following hydration, 250 μL of the T24 cell suspension was added to the top portion of each well with a final cannflavin A concentration of 2.5 μM. Seven hundred microliters of McCoy’s 5A medium containing 10% FBS was added to the bottom portion of each well. After 24 h, media and cells that did not invade were removed from the inside of the insert with a dampened cotton swab. Wells were placed in methanol for 10 min and then transferred into a 3.5 g/L Crystal Violet in 2% ethanol solution for 10 min. Wells were then rinsed with dH_2_0 and left to dry overnight. Cells that invaded through the Matrigel were counted using an Olympus CKX41 light microscope. Percent invasion was calculated by dividing the number of cells invaded in each condition by the number of cells that migrated in the control.

### Assessment of synergism, additivity, or antagonism

Synergies between cannflavin A and gemcitabine, cisplatin, or a combination of gemcitabine/cisplatin were studied using a checkerboard assay in T24 and TCCSUP cells. Synergy was also assessed between cannflavin A and Δ^9^-tetrahydrocannabinol, cannabidiol, cannabivarin, or cannabichromene. Briefly, the synergy assay was performed with 3000 cells/well in 96-well plates with a final volume of 100 μL per well. Drug concentrations ranged from 0 to 10 µM for the cannabinoids and up to 100 µM for the other drugs. Fluorescence was quantified as described before using AlamarBlue**®** after 48-h treatment. The analysis was performed using SynergyFinder 2.0 (Ianevski et al. 2020a), where the Bliss independence drug interaction model was used. Drug combination responses were also plotted as 3D synergy maps to assess the potential synergy, antagonism, or additive behaviors of the drug combinations. These maps provide visual representations of synergy and identified the concentrations at which the drug combinations had maximum effect on cell viability. The summary synergy represents the average excess response due to drug interactions. A synergy score of <− 10 was considered as antagonistic, a range from − 10 to + 10 as additive and > +10 as synergistic (Ianevski et al. 2020a, b).

### Statistical analysis

Statistical analysis was completed using GraphPad Prism. All error bars are representative of mean ± SEM. Unpaired Student’s *t*-tests were performed for analysis of two independent groups. One-way ANOVA with Tukey’s post hoc test was used to assess multi-group comparisons. *p* values are reported as follows: *p < 0.05, **p < 0.01, ***p < 0.001.

### Structure drawing

Schematic of the cannflavin A structure was done using the online ChemDraw JS tool, using the SMILES information found on Cayman Chemical’s cannflavin A datasheet: OC1=C(C/C=C(C)/CC/C=C(C)/C)C(O)=C(C(C=C(C2=CC=C(O)C(OC)=C2)O3)=O)C3=C1.

## Results

### Effect of individual drugs on cell viability

**Fig. 1:**
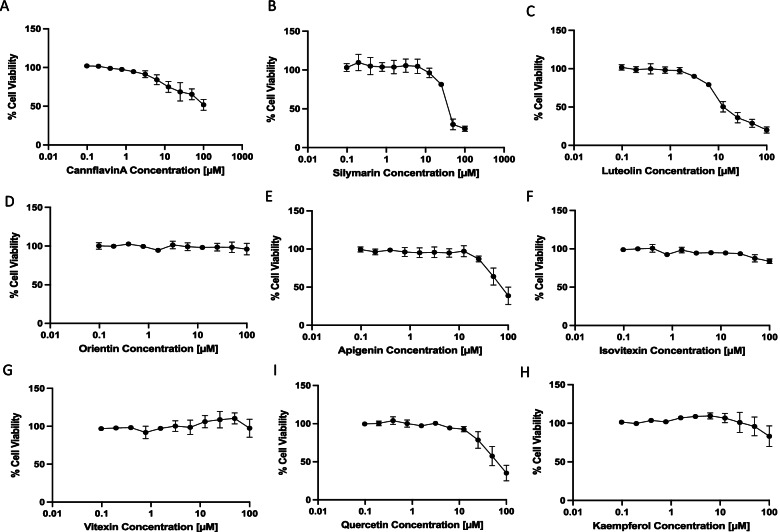
Effects of flavonoids on cell viability. Cell viability in T24 cells was assessed after 48-h treatment with **A** cannflavin A, **B** silymarin, **C** luteolin, **D** orientin, **E** apigenin, **F** isovitexin, **G** vitexin, **H** kaempferol, and **I** quercetin. The results indicate that flavonoids from cannabis induce variable effects, dependent on both the individual flavonoid and their concentrations. Results are means ± SEM of at least 3 independent experiments

**Fig. 2:**
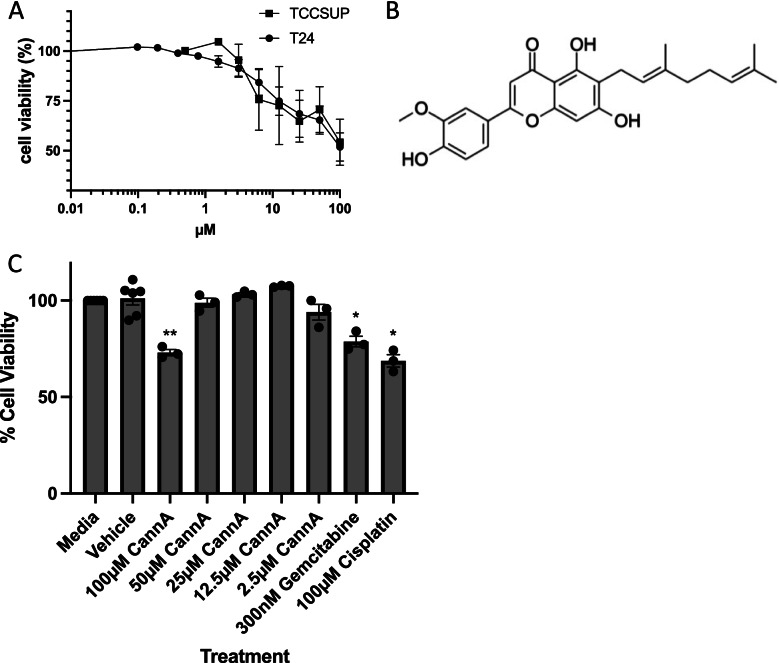
Effects of cannflavin A on cell viability. **A** Cell viability was assessed after 48-h treatment with cannflavin A in T24 and TCCSUP cells. The results from Fig. 1A in T24 cells were used to compare with the TCCSUP cell line. The results indicate that both transitional cell carcinomas are sensitive to cannflavin A in a similar fashion. **B** Schematic representation of the structure of cannflavin A. **C** Effects of cannflavin A on the viability of primary bladder epithelial cells. The results indicate that cannflavin A becomes cytotoxic at a concentration of 100 μM, but concentrations below this level are not cytotoxic. Results are means ± SEM of at least 3 independent experiments

The effects of the flavonoids cannflavin A, silymarin, luteolin, orientin, apigenin, isovitexin, vitexin, kaempferol, and quercetin on the cell viability of T24 bladder cancer cells were assessed (Fig. 1A–I). Cannflavin A (Fig. 1A), silymarin (Fig. 1B), luteolin (Fig. 1C), apigenin (Fig. 1E), and quercetin (Fig. 1I) showed the greatest concentration-dependent decreases in cell viability with approximately 49%, 76%, 80%, 61%, and 65% cell death, respectively. The cytotoxic effects of flavonoids have been reported in various systems, but since few plants have cannflavins in their tissues, there are not any data regarding its cytotoxic potential. We decided to further characterize the effects of cannflavin A in bladder cancer cells. To ensure the effects observed were not limited to a single cell line, cannflavin A data in T24 cells (Fig. 1A) was compared cell viability results in TCCSUP cells side by side (Fig. 2A). Cannflavin A displayed an IC_50_ of 15 µM and 8 µM in TCCSUP and T24 cells, respectively, after 48-h treatment. The effect of various concentrations of cannflavin A was also assessed in non-tumorigenic human epithelial bladder cells to determine the specificity of toxicity toward cancer cells vs normal epithelial bladder cells. The results show that cannflavin A concentrations ranging from 2.5 to 50 μM do not induce significant cytotoxicity compared to their vehicle control, while known chemotherapeutic treatments (gemcitabine and cisplatin) significantly reduce cell viability in the non-tumorigenic cell line (Fig. 2C). Cytotoxic effects of cannflavin A were observed at higher concentrations (100 μM). The maximum concentration used in our subsequent experiments was 50 μM.

### Effects of cannflavin A on apoptosis

**Fig. 3:**
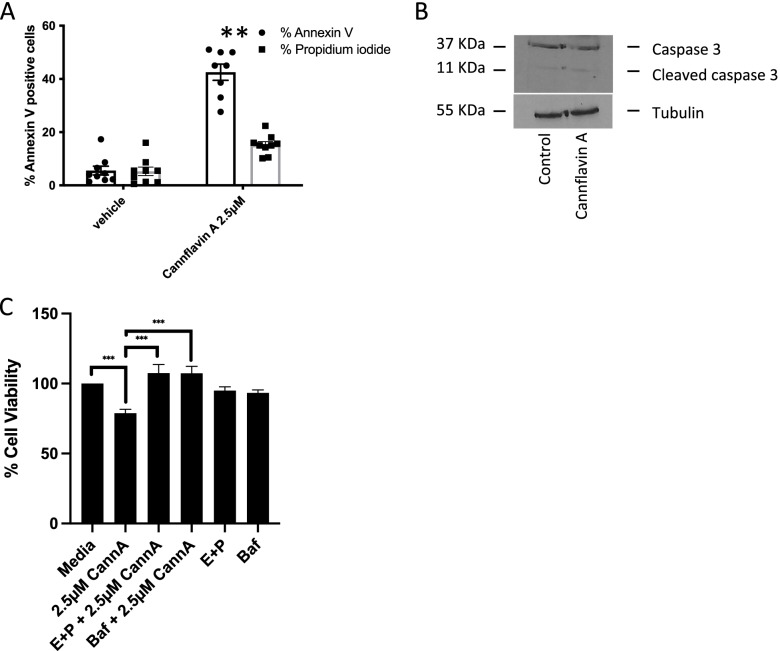
Effects of cannflavin A on apoptosis and autophagy. Cells were treated for 24 h for apoptosis or 6h for autophagy with either the methanol vehicle or cannflavin A. **A** Histogram showing the percentage of annexin V–labeled cells and % cells stained for propidium iodide, indicative of apoptosis. Cells were counted from three random fields of view on a fluorescence microscope. **p < 0.01, *n* of at least 8. **B** Western blotting analysis was performed using an anti-caspase 3 antibody, and β-tubulin was included as a loading control. The results indicate activation of caspase 3. Figure is a representative blot of *n* = 3 experiments. **C** Histogram showing the percentage of cell viability following 24-h cannflavin A treatment in T24 cells in the presence or absence of autophagy inhibitors (100 nM bafilomycin A or the combination of 10 μg/ml E-64d (aloxistatin) and 10 μg/ml (pepstatin A)). The inhibitors were able to revert the cytotoxic effects of cannflavin A. ***p<0.001, *n* of at least 3

Our group and others have recently shown that compounds from cannabis, like THC, CBD, and CBC, among others, can induce apoptosis in bladder cancer cells (Anis et al. 2021). Following a 24-h treatment of cells with a concentration of cannflavin A at which we did not detect significant changes in cell viability (2.5 µM), cannflavin A was shown to induce apoptosis. Our results show annexin V labeling of 42.5% ± 4.5 in T24 cells following cannflavin A treatment (Fig. 3A). Propidium iodide-labeled cells following cannflavin A treatment showed a slight increase that did not reach significance compared to the vehicle control. We then investigated the potential involvement of caspase 3 in the induction of apoptosis by cannflavin A and observed light cleavage of caspase 3 following ligand treatment for 24 h at a concentration of 2.5 µM (Fig. 3B).

### Effects of cannflavin A on autophagy

Many stimuli that ultimately cause cell death also trigger autophagy, which usually manifests well before apoptosis dismantles the cell. Rapid induction of autophagy reflects the instinct of the cell to adapt to stress and is followed by the activation of cell death pathways in response to multiple external including anticancer agents (Mariño et al. 2014). The effects of cannflavin A on cell viability in the presence or absence of bafilomycin (an inhibitor of autophagosome-lysosome fusion) or E-64d and pepstatin A (inhibitors of the degradation of autophagic cargo inside autophagolysosomes) were assessed (Fig. 3C). Cannflavin A induced a significant decrease in cell viability that was reversed in the presence of the autophagy inhibitors bafilomycin A and the combination of E-64d and pepstatin A suggesting a potential role of autophagy in the cytotoxic effects observed with cannflavin A in bladder cancer cells.

### Effects of cannflavin A on invasion

**Fig. 4:**
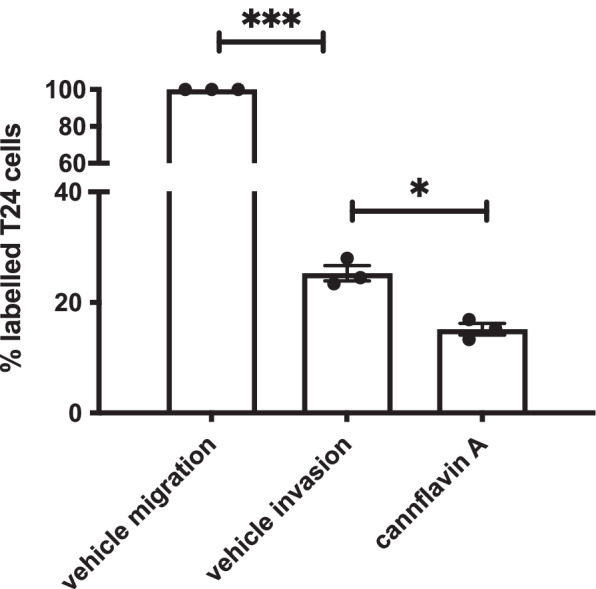
Effects of cannflavin A on invasion. Histogram summarizing Matrigel invasion assays using T24 cells in the presence of either the vehicle control or cannflavin A in comparison to vehicle-treated migration. The results indicate that cannflavin A may suppress the invasive ability of T24 cells. Results represent the means ± SEM of 3 experiments. *p < 0.1, ***p <0.001

In addition to its cytotoxic effects, we evaluated the potential for cannflavin A to reduce invasion of the high-grade and invasive T24 cells. T24 cells were seeded into Matrigel invasion chambers and treated with cannflavin A for 24 h. We then compared the results of the Matrigel invasion chambers between vehicle control and cannflavin A treatment. Our results indicate that T24 cells can invade the Matrigel and that cannflavin A treatment reduced their invasion (Fig. 4). In our control conditions, 25.3% of cells could invade the Matrigel. Following treatment of T24 cells with 2.5 μM of cannflavin A for 24 h, only 15.1% of cells could invade the Matrigel.

### Assessment of synergy between cannflavin A and chemotherapeutic agents

**Fig. 5:**
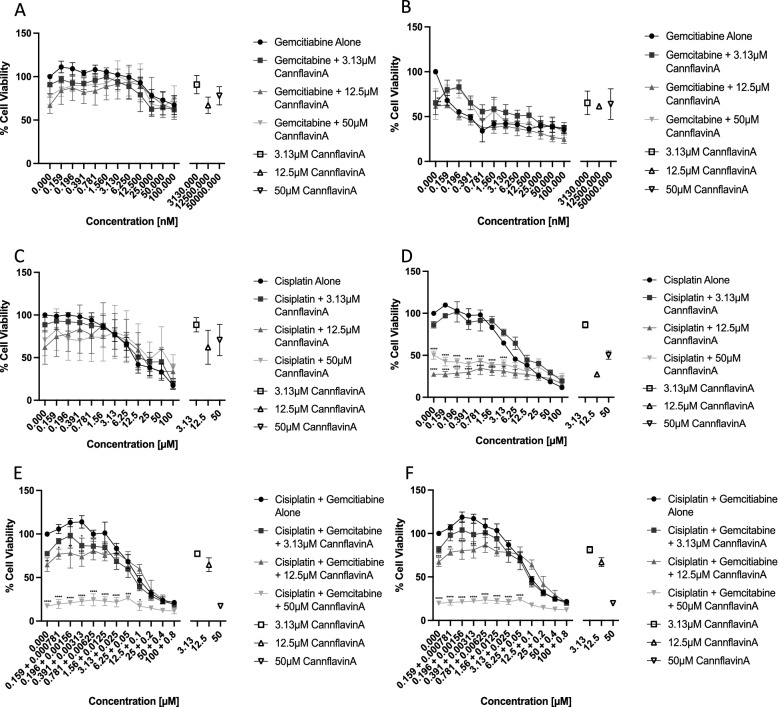
Assessment of synergy between cannflavin A and chemotherapeutic agents. Cell viability of T24 (**A**, **C**, **E**) and TCCSUP (**B**, **D**, **F**) cells following treatment with gemcitabine (**A** and **B**) cisplatin (**C** and **D**) or cisplatin/gemcitabine (**E** and **F**) combined with 3.13, 12.5, or 50 μM cannflavin A. The statistics indicate the significance of the addition of the various concentrations of cannflavin A compared to treatment with chemotherapeutic agents alone. In each graph, the effect of cannflavin A at 3.13, 12.5, or 50 μM cannflavin A is indicated by open data points (on the right of the graph). Results represent the means ± SEM of 3 experiments. *p<0.05, **p<0.01, ***p<0.001, ****p<0.0001

Gemcitabine-cisplatin treatment is a chemotherapeutic regimen used in bladder cancer, which prompted us to test the effects of cannflavin A co-treatment with gemcitabine (G), cisplatin (C), or the combination of GC on cell viability. Supplemental Fig. 1 shows the 3D synergy maps (Ianevski et al. 2020a) of the combinations tested. Our results indicate that depending on the concentration of the agents used, a variety of effects can occur, from antagonism to additivity or synergy. Table 1 shows the top and bottom 3 concentration combinations that generated the highest or lowest levels of interaction for cannflavin A with the chemotherapeutic agents. A score <− 10 is likely antagonistic; between − 10 and + 10 is likely additive; > +10 is likely synergistic, according to the synergy analysis model (Ianevski et al. 2020a). Our results identified some low levels of synergy between cannflavin A and cisplatin (maximum synergy score of around 14; 14% more than expected), but higher levels with gemcitabine (max synergy score of 32; 32% more than expected). The combination of cannflavin A with GC resulted in intermediate levels of synergy (max synergy score of 22) (Table 1). Most concentrations tested displayed additive effects, while some showed antagonism. Currently, statistical analysis of the synergy maps is not feasible using the SynergyFinder 2.0 tool. Therefore, to allow for statistical analysis, concentration curves of the chemotherapeutic agents were plotted with combinations of 3.13, 12.5, or 50 μM cannflavin A (Fig. 5), based on the results from the synergy grids demonstrated in Supplemental Fig. 1. First, no significant differences were observed with the addition of cannflavin A with gemcitabine in either T24 or TCCSUP cells (Fig. 5A, B) or with cisplatin in T24 cells (Fig. 5C). Cannflavin positively increased the cytotoxic effects of cisplatin in TCCSUP cells. In these cells, the combination of cannflavin A at concentrations of 12.5 and 50 μM significantly increased the cytotoxic effects between the concentrations of 0.156–3.13 μM of cisplatin (Fig. 5D), while at higher concentrations, no further cytotoxicity was observed. We also compared the combinations of cannflavin A and the combination of the chemotherapeutic agents gemcitabine and cisplatin at a ratio of 125:1. In T24 cells, the addition of 50 μM cannflavin A to the combination of cisplatin and gemcitabine was significantly different from the chemotherapeutic agents combination alone from 0.159 to 12.5 μM (of cisplatin); however, these effects are unlikely to be additive as the values are similar to 50 μM cannflavin A alone (Fig. 5E, inverted open triangle). Significant effects were also observed in T24 cells for the combinations of 12.5 μM cannflavin A and lower concentrations of the chemotherapeutic agent combination (0.159–1.56 μM cisplatin) (Fig. 5E). In TCCSUP cells, the cisplatin/gemcitabine combination (0.156–1.56 μM of cisplatin) with 12.5 μM cannflavin A and 1.59–6.25 μM with 50 μM cannflavin A (Fig. 5F) values were significant; however, the effects with 50 μM cannflavin A are once again unlikely to be additive as the values are similar to 50 μM cannflavin A alone (Fig. 5F, inverted open triangle).

**Table 1:** Highest and lowest levels of interaction between cannflavin A and gemcitabine and/or cisplatin in T24 cells

Score	CannA (μM)	Cisplatin (μM)	Score	CannA (μM)	Gemcitabine (nM)	Score	CannA (μM)	Gemcitabine (nM): cisplatin (μM)
*− 29.53*	50	6.25	*− 32.39*	1.25	25	*− 30.87*	0.625	62.48:7.81
*− 33.77*	50	12.5	*− 35.04*	1.25	12.5	*− 32.83*	12.5	17.81:0.97
*− 35.37*	100	50	*− 39.66*	1.25	6.25	*− 36.04*	12.5	15.6:1.95
9.72	1.56	0.2	**16.96**	2.5	50	**18.79**	6.25	62.48:7.81
**13.29**	100	0.39	**21.35**	0.625	50	**19.52**	25	250:31.25
**14.69**	25	0.39	**32.80**	0.625	25	**22.54**	3.13	250:31.25

A score < − 10 is likely antagonistic (italic); between − 10 and + 10 is likely additive (normal text); > +10 is likely synergistic (bold)

### Assessment of synergy between cannflavin A and other cannabinoids

**Fig. 6:**
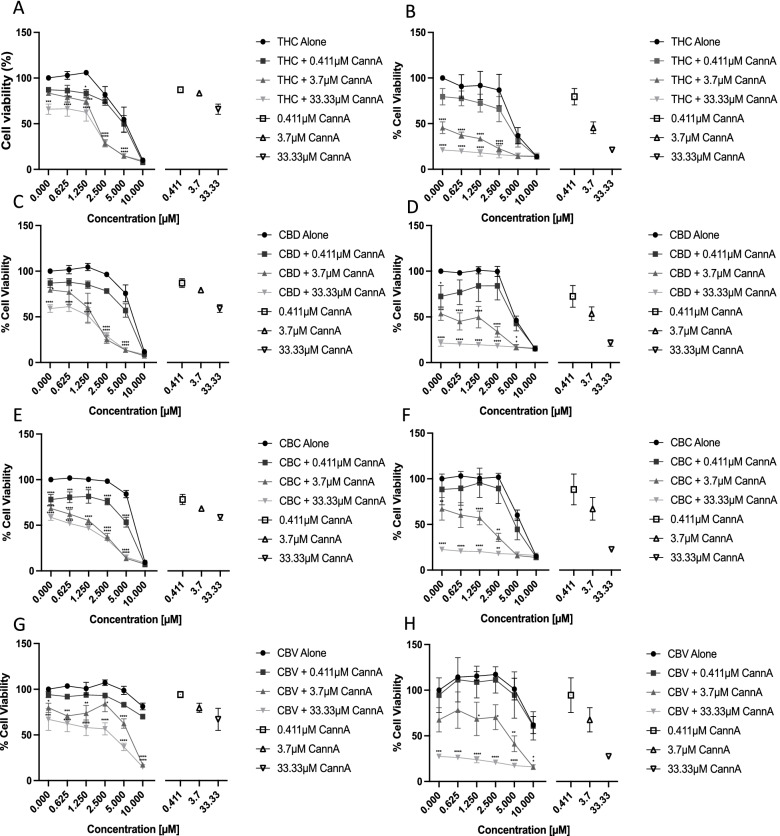
Assessment of synergy between cannflavin A and cannabinoids. Cell viability of T24 (**A**, **C**, **E**, **G**) and TCCSUP (**B**, **D**, **F**, **H**) cells following treatment with Δ9-tetrahydrocannabinol (**A** and **B**), cannabidiol (**C** and **D**), cannabichromene (**E** and **F**), or cannabivarin (**G** and **H**) combined with 0.411, 3.7, or 33.33 μM cannflavin A. The statistics indicate the significance of the addition of the various concentrations of cannflavin A compared to treatment with cannabinoids alone. In each graph, the effect of cannflavin A at 0.411, 3.7, or 33.33 μM cannflavin A is indicated by open data points (on the right of the graph). Results represent the means ± SEM of 3 experiments. *p<0.05, **p<0.01, ***p<0.001, ****p<0.0001. Combinations with 3.7 and 33.33 μM cannflavin A were deemed most statistically significant

In cannabis, different levels of the various components would be present together in the plant and their actions through various targets could be complementary and/or synergistic. While the results of the combinations of cannflavin A with gemcitabine or cisplatin on cell viability were largely within the additive range (− 10 to + 10, as seen in Table 1 and Supplemental Fig. 1), the combination of cannflavin A with cannabinoids produced much larger effects. As described earlier, some believe that cannabinoids represent a potential new drug class that could be used therapeutically for bladder cancer. We tested the effects of the combination of cannflavin A with Δ^9^-tetrahydrocannabinol, cannabidiol, cannabichromene, or cannabivarin (Fig. 6, Supplemental Fig. 2). Generally, the synergy scores (synergy is indicated by scores above + 10) were higher than what observed with the chemotherapeutic agents gemcitabine or cisplatin (Table 2). High synergy scores ranging above 40 were observed in most combinations, except for the combination of cannflavin A and cannabidiol (synergy score of approximately 25). To allow for statistical analysis, we tested the effects of the combination of 0.411, 3.7, or 33.33 μM cannflavin A with Δ^9^-tetrahydrocannabinol, cannabidiol, cannabichromene, or cannabivarin (Fig. 6). In T24 cells, both 3.7 and 33.33 μM cannflavin A combined with THC (Fig. 6A), CBD (Fig. 6C), and CBC (Fig. 6E) ranging from 0.625 to 5 μM, and CBV (Fig. 6G) ranging from 0.625 to 10 μM significantly decreased cell viability compared to the cannabinoids alone. Additionally, 0.411 μM cannflavin A in combination with CBC was significant for CBC concentration ranging from 0.625 to 5 μM (Fig. 6E). These effects are greater than the cannflavin A treatment alone as indicated by the open trial data points represented in the right portion of the graph. In TCCSUP cells, both 3.7 and 33.33 μM cannflavin A combined with THC ranging from 0.625 to 2.5 μM (Fig. 6B), CBD ranging from 0.625 to 5 μM (Fig. 6D), CBC ranging from 0.625 to 2.5 μM (Fig. 6F), and CBV ranging from 0.625 to 10 μM (Fig. 6H) significantly decreased cell viability compared to the cannabinoids alone. However, the combination of cannabinoids with 33.33 μM cannflavin A is unlikely to be additive as this concentration of cannflavin A alone is similar to the data point represented by the open inverted triangle data point on the far right of the graphs.

**Table 2:** Highest and lowest levels of interaction between cannabinoids and Δ9-THC, CBD, CBC, or CBV in T24 cells

Score	CannA (μM)	THC (μM)	Score	CannA (μM)	CBD (μM)	Score	CannA (μM)	CBC (μM)	Score	CannA (μM)	CBV (μM)
− 4.02	100	10	− 7.47	0.41	2.5	− 5.01	100	10	− 2.63	0.41	1.25
− 1.38	0.14	0.62	− 5.70	100	10	− 2.74	33.33	10	− 2.30	3.7	1.25
− 0.35	33.33	10	− 4.00	33.33	10	− 0.95	11.11	10	− 1.67	3.7	2.5
**64.85**	11.11	5	**25.45**	11.11	5	**46.64**	33.33	5	**41.88**	33.33	10
**68.23**	3.7	2.5	**25.48**	11.11	2.5	**51.67**	11.11	5	**42.37**	11.11	10
**68.48**	3.7	5	**33.37**	3.7	2.5	**57.66**	3.7	5	**42.77**	3.7	10

A score < − 10 is likely antagonistic (italic); between − 10 and +10 is likely additive (normal text); > +10 is likely synergistic (bold)

## Discussion

In this study, the effects of cannflavin A were tested for their potential cytotoxic effects in bladder cancer cells. Our results indicate that cannflavin A can reduce cell viability of human bladder transitional cell carcinoma cell lines. Compared to other flavonoids like silymarin, quercetin, or luteolin, cannflavin A displayed moderate cytotoxicity, and cannflavin A’s toxicity was specific to cancer cells when concentrations below 100 μM were used. We demonstrated that autophagy and apoptosis are induced following cannflavin A treatment and that caspase 3 is involved. Additionally, we showed that cannflavin A reduces the invasion of the highly invasive T24 cell line. Finally, we tested the synergistic effects of the combination of cannflavin A with gemcitabine-cisplatin chemotherapeutic agents as well as with cannabinoids. Our results showed that while some synergy is possible with gemcitabine or cisplatin, much higher levels of synergy occurred when cannabinoids like Δ^9^-tetrahydrocannabinol, cannabichromene, or cannabivarin are combined with cannflavin A. In recent years, several groups have begun exploring the potential of cannabinoids as anticancer agents, and this in various cancer types (Blasco-Benito et al. 2018; López-Valero et al. 2018a, b), including bladder cancer (Anis et al. 2021; Chen et al. 2021).

We compared the efficacy of cannflavin A to other flavonoids also present in cannabis, several of which were previously shown to exert cytotoxic effects. The relative IC_50_ values observed for the cytotoxic effects of cannflavin A were determined to be 8 and 15 μM in TCCSUP and T24 transitional cell carcinoma cell lines, respectively. One issue with flavonoids is their relative lack of specificity, where non-cancer cells may also be affected by their exposure to flavonoids. Here, we show that at higher concentrations (100 μM), cannflavin also displayed significant toxicity toward primary bladder epithelial cells, but that concentrations below 50 μM were not toxic to primary epithelial bladder cells. These higher levels of cannflavin may not be reached when cannabis is consumed, which would reduce the potential for this agent to affect the normal epithelial in patients consuming cannabis products. Various methods including intravesical therapy, for example, could allow to avoid systemic treatment and permit to choose concentrations that would selectively treat bladder cancer in vivo with minimal effect on normal cells. As for any chemotherapeutic agent, the potential benefits of agents like cannflavin A versus their potential toxicity will need to be evaluated more attentively in vivo to assess whether it would represent a viable new therapeutic agent for the treatment of cancer.

We also investigated potential mechanisms by which these cytotoxic effects could occur. Our results indicate that apoptosis is induced by cannflavin A and that caspase 3 is involved. Additionally, it has been previously demonstrated that flavonoids may induce autophagy (Pang et al. 2021), so we tested the potential of cannflavin A to induce this pathway. Bafilomycin A1, which blocked the cytotoxic effects of cannflavin A in our study, has been previously shown to disrupt autophagic flux by inhibiting both V-ATPase-dependent acidification and autophagosome-lysosome fusion (Mauvezin and Neufeld 2015). The lysosomal inhibitors E-64d and pepstatin A also displayed an effect at blocking cannflavin A’s cytotoxicity. Our results indicate that autophagy may participate in the process leading to bladder cancer cells death. Interestingly, not only are death signaling pathways activated, but other signaling pathways linked to migration and invasion are also altered with cannflavin A treatment. The invasion of high-grade and invasive T24 transitional cell carcinoma cells was reduced following treatment with cannflavin A at a concentration that did not alter cell viability. These results suggest that cannflavin A could potentially reduce invasion of bladder cancer.

It has been reported that dietary consumption of various plant flavones offers neuroprotective, antioxidant, and anticancer properties in several animal models. While several flavones and their biosynthetic pathways have been extensively studied in some plants, little is known about some of these compounds found in cannabis. For example, cannflavins A and B appear to be specific to cannabis (Vanhoenacker et al. 2002). These compounds have been shown to exert anti-inflammatory, anti-parasitic, and neuroprotective effects (Rea et al. 2019; Barrett et al. 1985, 1986; Eggers et al. 2019; Ibrahim et al. 2010), but only one study, using a derivative of cannflavin B, has demonstrated anticancer effects (Moreau et al. 2019). In vitro results showed an increase in apoptosis in two pancreatic cancer cell lines treated with concentrations of FBL-03G (or caflanone). Local and metastatic tumor progression was delayed in pancreatic cancer animal models, leading to an increase in survival. Our study demonstrates that these anti-cancer properties may extend to other members of the cannflavin family and may be applicable to bladder cancer therapy.

Multiple studies have demonstrated the ability of chemotherapeutic agents used for the treatment of bladder cancer, like gemcitabine and cisplatin, to act synergistically with other compounds and produce enhanced anti-cancer effects (Ma et al. 2010; Mey et al. 2006; Rabenstein et al. 2017). We identified some lower levels of synergy between cannflavin A and gemcitabine or cisplatin. The suggestion of adding new compounds to currently prescribed chemotherapeutic agents to improve outcomes is increasingly more common. For example, kaempferol has been suggested to be added with 5-fluorouracil as it displayed synergistic anti-proliferative effects and re-sensitized resistant cells to chemotherapeutic agents in therapy-resistant colon cancer cells (Riahi-Chebbi et al. 2019; Li et al. 2019). When quercetin was added to gemcitabine increased apoptosis in gemcitabine-resistant cancer cells was observed (Liu et al. 2020). Here, we observed that cannflavin A may modestly alter the efficacy of chemotherapeutic agents, depending on the concentration used. These results remain to be validated in vivo; however, they provide an indication of the range of concentrations that could be required to generate effects in combination therapy involving this compound. One aspect that is more striking is how cannflavin A may synergize with cannabinoids to increase the cytotoxic effects on bladder cancer cells. Our results suggest that combining cannflavin A with more common compounds from cannabis like cannabidiol or Δ^9^-tetrahydrocannabinol or even other cannabinoids like cannabichromene or cannabivarin may improve the overall efficacy of the cytotoxic treatment. Other studies have looked at the combination of cannabinoids (Δ^9^-tetrahydrocannabinol + cannabichromene) and have found synergistic effects in bladder cancer (Anis et al. 2021), but our study is the first to show synergistic cytotoxic effects between a flavonoid and cannabinoid in bladder cancer cells.

## Conclusions

In recent years, several studies have attempted to characterize how cannabinoids and other compounds present in the cannabis plant work together. Some have suggested an entourage effect, where the various components of the plant work together to produce larger, synergistic effects either via the same target or through activation of multiple complementary mechanisms. Comparison of pure cannabinoids and botanical extracts has shown that botanical preparations produce larger anti-tumor responses in vitro and in vivo, versus Δ^9^-tetrahydrocannabinol alone (Blasco-Benito et al. 2018). Unfortunately, the terpenes suggested as potential mediators of the synergy were not identified in that study, indicating that potentially other compounds present within the extract could mediate the effects observed. The levels of flavonoids are rarely examined within cannabis extracts, and it is possible that they could contribute to the overall effects as well. In bladder cancer, the effects of cannabis or compounds isolated from cannabis have not been extensively studied. A study recently demonstrated that the combination of Δ^9^-tetrahydrocannabinol and cannabichromene produced synergistic effects in a bladder cancer model (Anis et al. 2021), while another focused on the effects of cannabidiol and their potential formulation within nanoparticles to treat bladder cancer (Chen et al. 2021). Here, we show that other compounds from cannabis, like cannflavin A, may also induce beneficial cytotoxic and synergistic effects on bladder cancer cells. Our results also showed the ability of cannabinoids, other than Δ^9^-tetrahydrocannabinol, to produce synergistic effects when combined with the flavonoid cannflavin A. While these results remain to be validated in in vivo models and in human clinical trials, our study is the first to investigate the cytotoxic effects of cannflavin A in the treatment of bladder cancer and demonstrate its potential benefits. More investigation is needed to determine how cannabinoids and other compounds from cannabis like cannflavin A could be used therapeutically in the treatment of cancers and whether they could be used similarly to cannflavin B, alone, or in combination with other chemotherapeutic agents.

## Supplementary Information

**Additional file 1: Supp Fig. 1.** Assessment of synergy between cannflavin A and chemotherapeutic agents. **Supp Fig. 2.** Assessment of synergy between cannflavin A and cannabinoids.

## Data Availability

The datasets used and/or analyzed during the current study are available from the corresponding author on reasonable request.

## Abbreviations

TCC: Transitional cell carcinoma
MVAC: Methotrexate, vinblastine sulfate, doxorubicin hydrochloride (Adriamycin), and cisplatin
THC: Tetrahydrocannabinol
CBD: Cannabidiol
CBC: Cannabichromene
G: Gemcitabine
C: Cisplatin
