# Supplementary material for: Anti-cancer properties of cannflavin A and potential synergistic effects with gemcitabine, cisplatin, and cannabinoids in bladder cancer
Source: J Cannabis Res. 2022 Jul 22;4:41. doi: 10.1186/s42238-022-00151-y (PMC9306207; doi:10.1186/s42238-022-00151-y)
Supplement: Supplementary file 1 — Additional file 1: Supp Fig. 1. Assessment of synergy between cannflavin A and chemotherapeutic agents. Supp Fig. 2. Assessment of synergy between cannflavin A and cannabinoids. [file 42238_2022_151_MOESM1_ESM.pdf]

Supplemental Figure 2

T24 TCCSUP

Cannflavin A

Δ9-THC

Cannabidiol

Cannabichromene

Cannabivarin

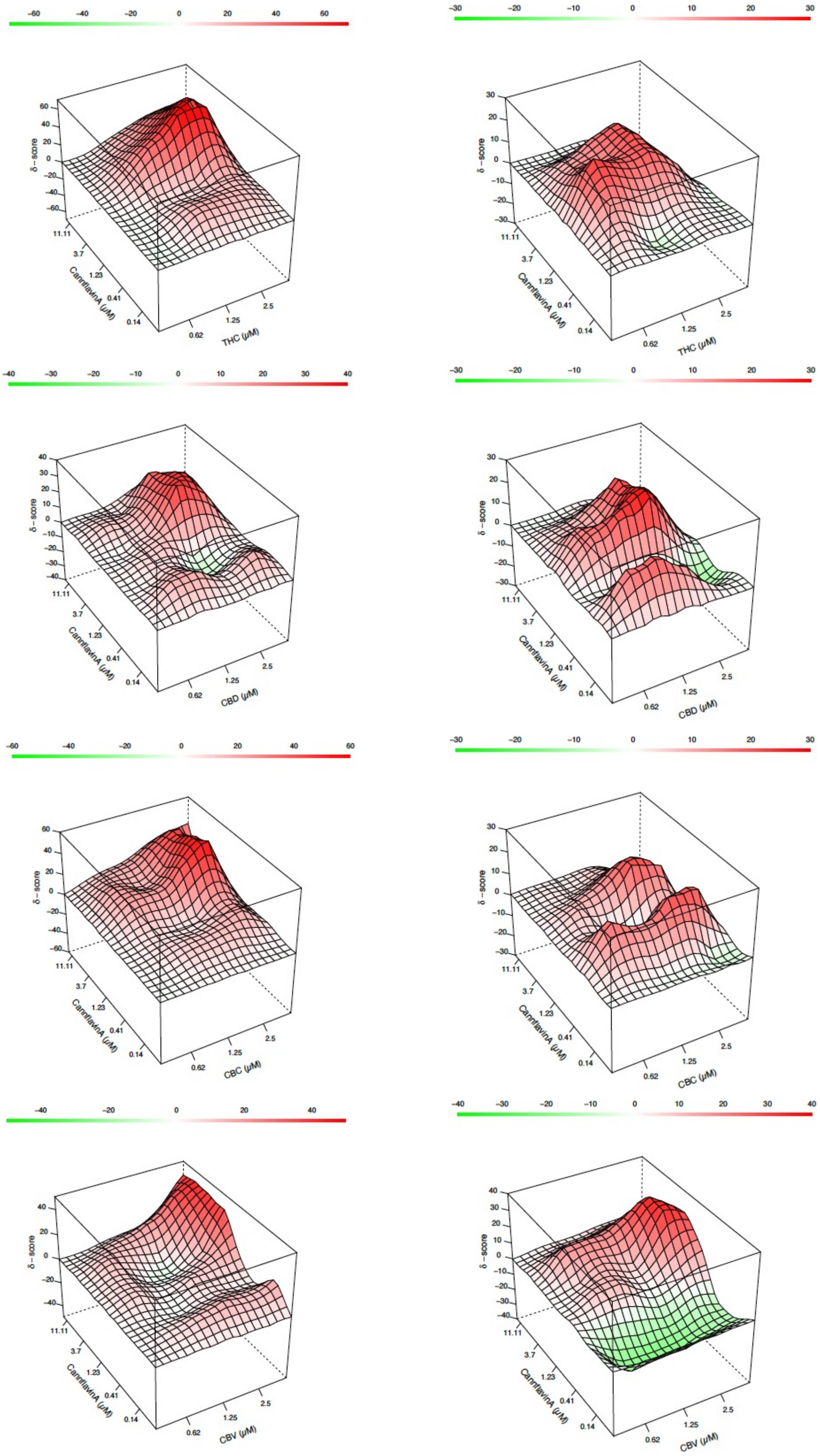

SUPP FIG. 2. Assessment of synergy between cannflavin A and cannabinoids
3D synergy landscapes for the combinations of increasing concentrations of cannflavin A with THC, cannabidiol, cannabichromene or cannabivarin in T24 and TCCSUP cells.
